# Greening Gastroenterology: Strategies to Reduce the Carbon Footprint in Clinical Practice

**DOI:** 10.7759/cureus.103520

**Published:** 2026-02-13

**Authors:** Heewon Yoon, Sara Razi, Moses Oigara, Supreeth Sujithkumar, Abhilasha Katoch, Ashrit Alinkil, Gurinder Singh, Manju Rai

**Affiliations:** 1 Surgery, University of Central Lancashire, Preston, GBR; 2 Internal Medicine, Islamic Azad University of Tehran Medical Sciences, Tehran, IRN; 3 Pediatrics, Tenwek Hospital, Nairobi, KEN; 4 Internal Medicine, All India Institute of Medical Sciences (AIIMS), Bhopal, IND; 5 Gastroenterology, Duke University School of Medicine, Durham, USA; 6 General Medicine, Swansea Bay University Health Board, Swansea, GBR; 7 Clinical and Medical Sciences, Universidad Especializada de las Américas, Panama City, PAN; 8 Biotechnology, Shri Venkateshwara University, Gajraula, IND

**Keywords:** carbon footprint, gastroenterology, green endoscopy, medical waste management, sustainable healthcare

## Abstract

Climate change is an escalating global health concern, and healthcare delivery contributes meaningfully to environmental impact, with gastroenterology among the more resource-intensive clinical fields. This narrative review explores the environmental footprint of gastroenterology - especially endoscopic practices - and presents actionable strategies to reduce its carbon emissions without compromising care quality. Endoscopic procedures, high in energy consumption and medical waste generation, contribute substantially to the specialty’s carbon output. Additional contributors include pharmaceutical waste, inefficient waste management, and travel-related emissions from both patients and professionals. The review identifies several practical interventions, such as promoting combined procedures, reducing unnecessary biopsies, adopting reusable accessories, and implementing telemedicine and virtual conferencing to minimize travel. Sustainable disinfection technologies like plasma-activated gas and UVC light, alongside green pharmacy principles and antibiotic stewardship, are highlighted as promising innovations. Barriers to implementation - ranging from economic and regulatory constraints to clinician resistance - are critically examined, with recommendations to embed sustainability in clinical guidelines, staff training, and procurement practices. Real-world case studies underscore the feasibility and benefits of adopting sustainable gastroenterological practices across varied healthcare settings. Ultimately, the review calls for systemic transformation driven by standardized sustainability metrics, investment in green technologies, and equitable global collaboration. As climate change increasingly intersects with gastrointestinal health, greening gastroenterology is not only feasible but imperative for ensuring both environmental and patient well-being.

## Introduction and background

Climate change is now recognized as one of the most pressing global health threats of the 21st century, with wide-ranging consequences for human well-being, healthcare delivery, and planetary sustainability. The healthcare sector paradoxically contributes significantly to the very crisis it seeks to mitigate, emitting an estimated 4.4% of global greenhouse gases (GHGs) [1]. If considered as a nation, global healthcare would rank as the fifth-largest emitter of carbon dioxide equivalents (CO₂e), underscoring the urgent need for sector-wide decarbonisation [1]. Within this context, the field of gastroenterology occupies a uniquely resource-intensive niche, characterized by high procedural volumes, energy-demanding technologies, and considerable waste generation, particularly in the realm of endoscopic interventions.

The rising demand for gastrointestinal (GI) services, driven by aging populations, increased prevalence of digestive diseases, and advances in diagnostic and therapeutic technologies, has led to a proportional escalation in the specialty's environmental footprint. Endoscopic procedures, which form the backbone of modern gastroenterology, require stringent sterilization protocols, frequent use of single-use devices, and substantial energy inputs. These practices collectively contribute to high volumes of solid, liquid, and hazardous waste [2-3]. Moreover, travel-related emissions from patients, providers, and professional gatherings further exacerbate the specialty's environmental impact [4]. As climate change continues to affect public health, including rising rates of GI infections, malnutrition, and inflammatory diseases, it becomes imperative that gastroenterology not only adapts to these challenges but also plays an active role in mitigation.

**Figure 1:**
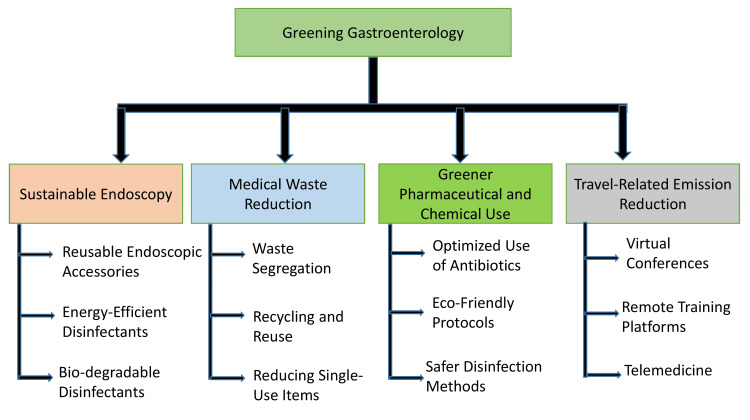
Key strategies for greening gastroenterology practice Figure created by author Gurinder Singh using Biorender

Recent years have seen growing awareness of the healthcare sector's environmental responsibilities, prompting calls for the adoption of sustainable practices across clinical disciplines. The concept of "green healthcare" has gained traction, advocating for environmentally conscious decision-making without compromising the quality, safety, or accessibility of patient care. In gastroenterology, this translates to reevaluating current practices, implementing waste reduction strategies, optimizing resource utilization, and embracing technological innovations that reduce carbon intensity. Several healthcare systems and academic institutions have already initiated sustainability programs, ranging from eco-friendly endoscopy suites to carbon-conscious conference planning, providing proof of concept for scalable, evidence-based solutions (Figure 1).

Despite this progress, the literature remains fragmented, and there is a paucity of comprehensive guidance tailored specifically to gastroenterology. To address this gap, this review synthesizes current knowledge on the environmental impact of gastroenterology practice and explores practical strategies for reducing its carbon footprint. The review focuses on evaluating the environmental impact of medical waste, pharmaceutical use, and travel-related emissions associated with diagnostic and therapeutic procedures. Furthermore, the review highlights actionable, evidence-based strategies, supported by real-world examples, that can be implemented to mitigate the carbon footprint of gastroenterology, thereby contributing to a more sustainable healthcare system. 

## Review

Methodology

This article is a narrative review synthesizing current evidence on the environmental impact of gastroenterology practice and strategies to reduce its carbon footprint. The review was conducted to provide a comprehensive, clinically relevant overview of sustainability-focused interventions applicable to diagnostic, therapeutic, and organizational aspects of gastroenterology, without employing formal quantitative synthesis or meta-analytic techniques.

A structured literature search was performed across major electronic databases, including PubMed/MEDLINE, Scopus, and Google Scholar, to identify relevant publications published between January 2009 and August 2025. Additional sources were identified through manual screening of reference lists from key articles, position statements, and consensus guidelines to ensure completeness.

The search strategy incorporated a combination of Medical Subject Headings (MeSH) terms and free-text keywords related to sustainability and gastroenterology. Commonly used MeSH terms and keywords included: "Gastroenterology", "Endoscopy", "Carbon Footprint", "Climate Change", "Sustainable Healthcare", "Medical Waste", "Green Endoscopy", "Environmental Impact", "Healthcare Sustainability", and "Telemedicine". These terms were combined using Boolean operators (AND/OR) to refine and broaden the search as appropriate.

Articles were considered eligible for inclusion if they addressed environmental sustainability, carbon emissions, waste generation, or resource utilization in gastroenterology or endoscopic practice; included original research studies, narrative or systematic reviews, position statements, consensus documents, or real-world case studies; were published in peer-reviewed journals and available in English; and had relevance to clinical practice, healthcare systems, or policy-level sustainability interventions.

Exclusion criteria included conference abstracts without full-text availability, editorials lacking substantive data or analysis, non-peer-reviewed opinion pieces, and studies unrelated to gastroenterology or gastrointestinal procedures. Articles focusing exclusively on non-clinical environmental topics without healthcare relevance were also excluded.

**Figure 2:**
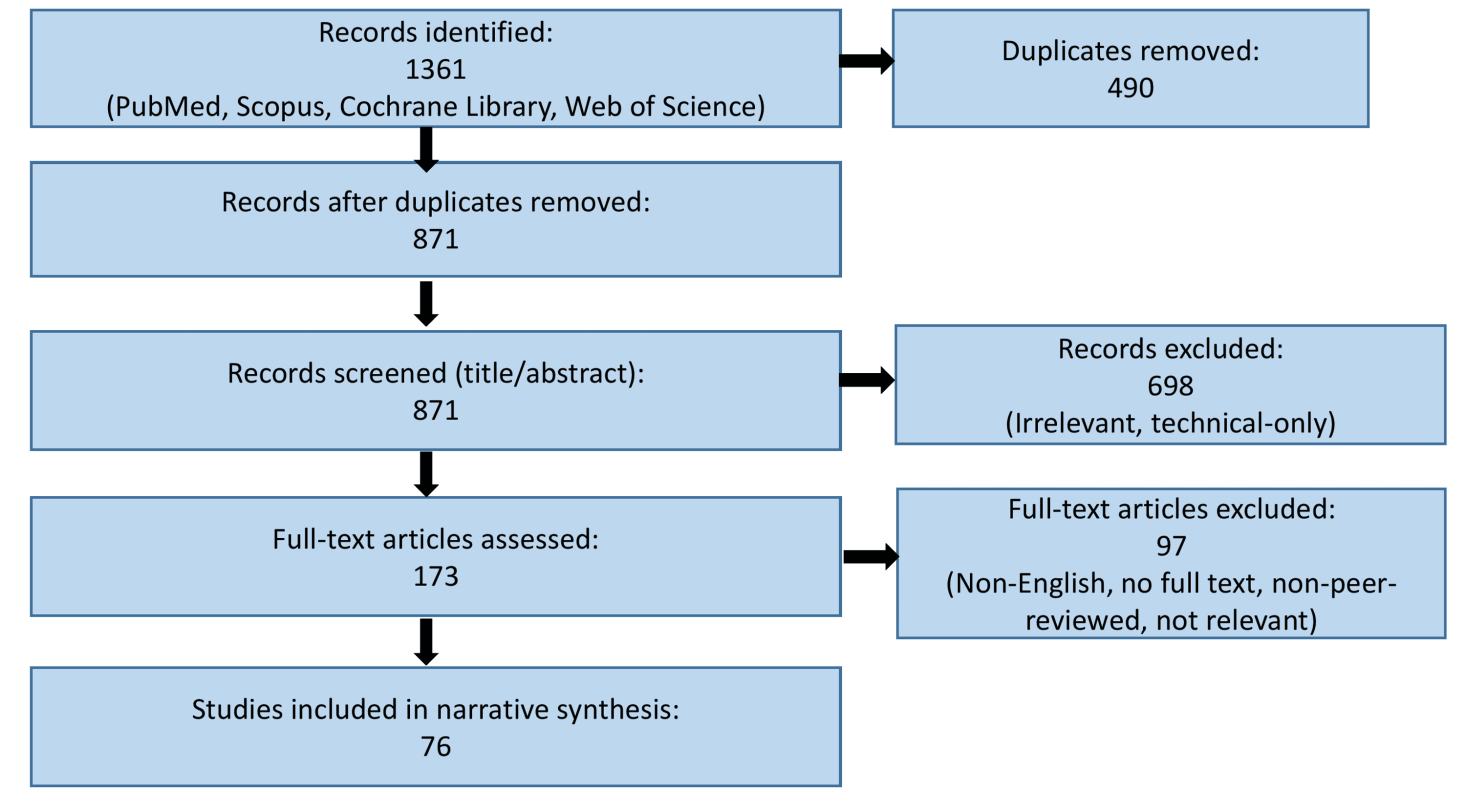
Flow diagram summarizing the study selection process for the included literature in this narrative review

Given the narrative nature of the review, data extraction was performed qualitatively. Key themes were identified, including carbon emissions from endoscopic procedures, medical and pharmaceutical waste, travel-related emissions, sustainable technologies, digital transformation, and barriers to implementation. Findings were synthesized descriptively to highlight trends, practical interventions, and context-specific considerations across diverse healthcare settings. No formal risk-of-bias assessment or statistical pooling of results was undertaken, consistent with the aims of a narrative review. Figure 2 presents a flow diagram illustrating the literature identification, screening, eligibility assessment, and inclusion process for studies considered in this review. Although elements of the Preferred Reporting Items for Systematic Reviews and Meta-Analysis (PRISMA) framework were applied to enhance transparency in literature identification and selection, this review was designed as a narrative synthesis rather than a formal systematic review, and therefore does not include quantitative pooling or risk-of-bias assessment.

Environmental impact of gastroenterology practice

Carbon Footprint of Endoscopic Procedures

Endoscopy, in particular, plays a prominent role in healthcare-related emissions. Endoscopy ranks among the top three medical specialties contributing to GHG emissions, primarily due to its high procedural volume, rigorous sterilization protocols, and reliance on energy-intensive equipment [5]. For every 100 GI endoscopic procedures, an estimated 303 kilograms of solid waste, 1385 liters of liquid waste, and 1980 kilowatt-hours of electricity are consumed, of which only 20% is recyclable [6]. A study by Sonaiya et al. (2024) estimated that endoscopic practices in the United States produce approximately 85,768 metric tons of CO₂-equivalent (tCO₂e) emissions annually [2]. The study found that the use of single-use endoscopes alone contributes approximately 1.37 kg of CO₂ per procedure, whereas reusable alternatives emit as little as 0.0017 kg of CO₂.

Furthermore, emissions from patient and staff travel, equipment manufacturing, and energy use underscore endoscopy as a critical area for targeted decarbonization efforts in clinical practice [7]. Another major contributor to the environmental burden of endoscopy units is the extensive use of packaged sterile water, both during procedures and in decontamination processes. The production, transportation, and disposal of these containers consume significant energy and resources [8].

Medical Waste in Gastroenterology

The environmental burden of gastroenterology is further compounded by the substantial volume of medical waste it generates. Vu et al. reported that 450 general GI procedures performed on 400 patients in a single endoscopy suite generated approximately 1398.6 kilograms of solid waste [9]. Of this, 1010.65 kg was designated as landfill waste, 336.34 kg as biohazard waste, and 51.57 kg as sharps. Similarly, a study from South Korea estimated that each endoscopic procedure generates an average of 1.34 kg of medical waste, including disposable suction tubing, packaging, gloves, and gowns [10]. Operating rooms and procedural areas are known to contribute disproportionately to overall healthcare waste, thereby exacerbating environmental, economic, and energy concerns [11].

Improper waste disposal contributes to environmental degradation, including groundwater contamination, methane production, and ecosystem disruption. Consequently, the implementation of sustainable waste management strategies has become imperative.

Pharmaceutical and Chemical Waste

Globally, pharmaceutical waste constitutes approximately 3% of the total waste generated by healthcare services [12]. The growing volume of pharmaceutical by-products has raised considerable concern regarding their disposal [13]. The improper handling and discarding of unused medications have become a mounting global concern [14]. This challenge is particularly pronounced in developing nations, where the management of hazardous pharmaceutical waste remains inadequate, and structured medication disposal programs are often absent [15]. For instance, a study revealed that practices related to the handling, storage, and disposal of medical waste were suboptimal when compared to standards observed in developed countries [16]. The management of both hazardous and general medical waste in these settings frequently falls short of internationally accepted safety and environmental guidelines.

Pharmaceutical waste can originate from a broad spectrum of activities within the healthcare system [13]. However, the rising consumption of pharmaceuticals has introduced additional health risks, primarily due to the increased discharge of pharmaceutical residues into the environment during both usage and disposal [17]. Inadequate management of such healthcare waste can pose significant risks not only to medical personnel and waste management staff but also to patients and the broader community [12]. Research has shown that limited awareness and poor segregation practices of medical waste can adversely affect the quality and safety of healthcare services [18]. A study demonstrated a statistically significant relationship between environmental factors and the overall efficiency of hospital waste management practices, including waste segregation, classification, collection, and storage processes [19]. The study found an overall pharmaceutical wastage rate of 3.68%, primarily due to expiry (92.05%), with supplies being the most wasted category and tablets and injectables having high wastage rates of 20.78% and 16.49%, respectively.

Travel-Related Emissions

Travel-related emissions, particularly those associated with professional meetings and patient visits, represent an often underestimated source of carbon output in gastroenterology. In-person attendance at conferences contributes significantly to GHG emissions, largely due to air travel. For instance, the 2019 American Society of Clinical Oncology (ASCO) in-person conference generated an estimated 37,251 metric tons of CO₂ equivalents [19]. In contrast, the 2020 virtual edition of the same conference produced only 115.36 metric tons [19-20]. Staff commuting accounts for approximately 4% of total greenhouse gas emissions within the NHS [8]. Pioche et al. (2024) reported that capsule endoscopy produced a total of 20 kg of CO₂ emissions, with the vast majority (18 kg CO₂) stemming from patient travel to the endoscopy unit, while the capsule device itself contributed only a negligible 0.04 kg CO₂ [21].

Strategies to reduce the carbon footprint in gastroenterology

Sustainable Endoscopy Practices

**Figure 3:**
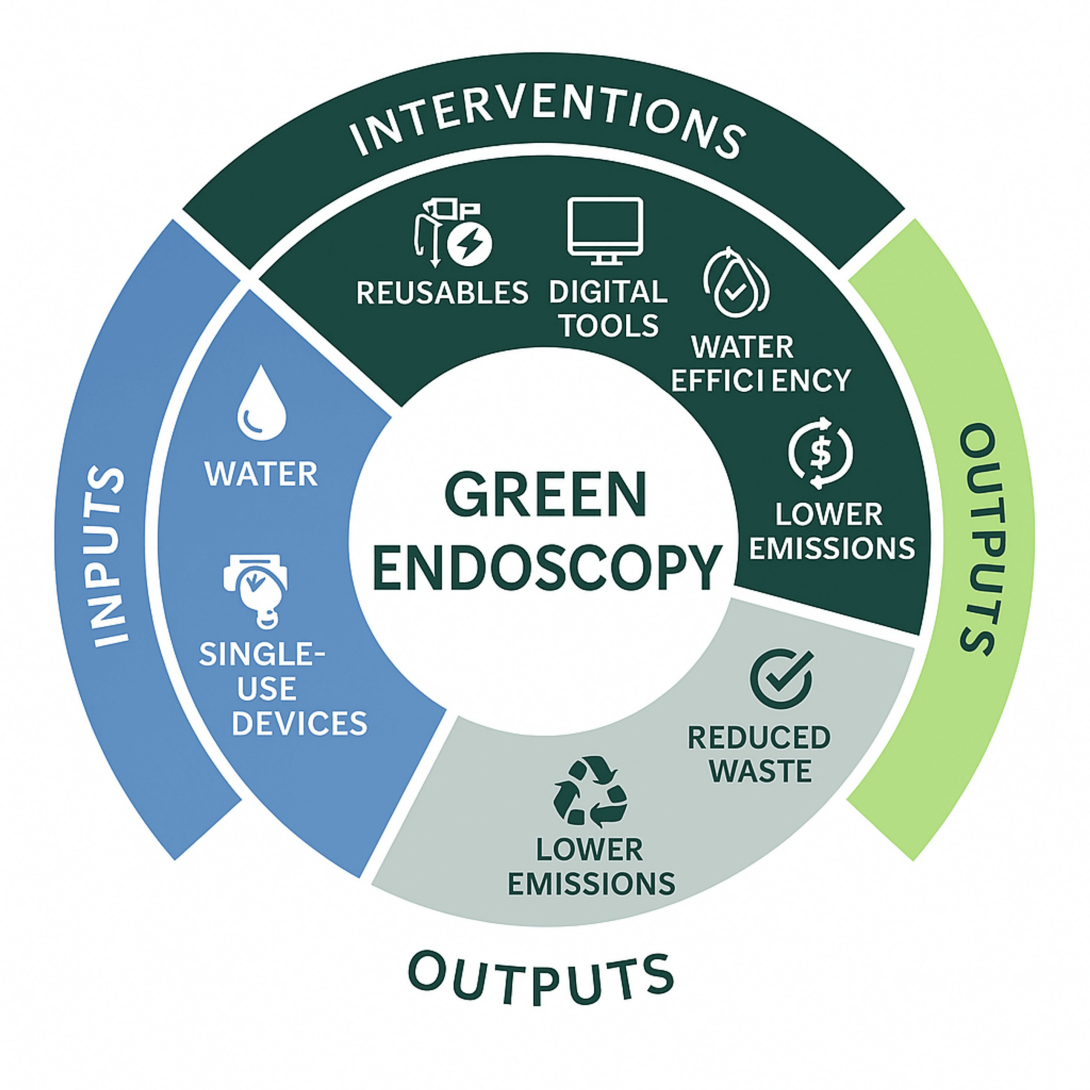
Circular model of green endoscopy This circular diagram illustrates the sustainable transformation of endoscopic practice by mapping the cycle from traditional inputs to green outputs. Inputs such as water and single-use devices are targeted by interventions - specifically, the adoption of reusable accessories, digital tools (e.g., telemedicine, electronic documentation), and water-efficient practices. These interventions collectively lead to environmentally favorable outputs, including reduced waste, lower greenhouse gas emissions, and a more resource-efficient model of care. Figure created by author Moses Oigara using Biorender

A growing emphasis on sustainability within healthcare has prompted the gastroenterology community to evaluate and reform endoscopy practices to minimize their environmental impact (Figure 3). One such strategy involves promoting combined upper and lower GI procedures on the same day, which has been associated with reduced carbon emissions [8]. Although comparative data remains limited, the assumption is that single-session bidirectional endoscopy reduces patient travel, lowers the usage of personal protective equipment (PPE), and conserves both water and energy resources. Consequently, performing combined endoscopy when clinically indicated is encouraged as an environmentally favorable practice [8].

Institutions are encouraged to perform internal audits and educate staff about the environmental impact of energy and resource-consuming production, transportation, and disposal cycle of sterile water. Sterile water, therefore, should be used judiciously, and leftover amounts may be repurposed, for example, for bedside cleaning, rather than discarded [8]. Improving the efficiency of the decontamination process is another critical intervention. Since over 90% of water use in endoscopy units occurs during decontamination, optimizing this process with machines that reduce energy and water consumption can significantly lower the carbon footprint [22]. In the absence of strict manufacturer guidelines, units may evaluate the safety of substituting sterile water with tap water for routine endoscopic cleaning [22].

Energy efficiency in endoscopic unit design is also essential. Given the high electricity demands of operating theatres, incorporating natural lighting, using energy-saving or automated lighting systems, and shutting down non-essential equipment can substantially reduce emissions [23]. Heating, ventilation, and air conditioning (HVAC) systems are the most energy-intensive components of hospital infrastructure [24]. Implementing HVAC energy setbacks to maintain only the essential pressure levels in endoscopy suites and utilizing technologies such as blockchain to automate and optimize energy distribution can further enhance sustainability [24].

In addition to physical infrastructure, promoting remote work for administrative staff can reduce emissions associated with commuting. Furthermore, virtual consultations for result communication or low-risk procedures offer an opportunity to reduce travel-related emissions without compromising patient care [22].

Reducing the volume of unnecessary endoscopic procedures and biopsies is a powerful sustainability measure. An estimated 40% of endoscopic procedures are considered unnecessary, and histological findings from routine biopsies rarely alter clinical management, influencing decisions in only about 17% of cases [8,21]. Inappropriate procedures and biopsies not only generate avoidable emissions but also strain healthcare resources. Institutions should develop triage protocols, adopt evidence-based guidelines, and consider non-invasive diagnostic alternatives when feasible [22,25]. Patient education has proven to be an effective intervention in this regard. For instance, virtual dyspepsia education led to a 40% reduction in gastroscopy rates in one study, highlighting its potential to reduce procedure volumes through informed decision-making [26].

Artificial intelligence (AI) offers promising applications in endoscopy by facilitating lesion risk stratification and minimizing unnecessary biopsies. Deep learning models have demonstrated the ability to distinguish high-risk anal intraepithelial lesions from low-risk counterparts, reducing the need for invasive interventions [27].

When biopsies are clinically necessary, reducing the number of collection pots can yield environmental benefits. It has been demonstrated that placing polyps smaller than 10 mm in a single collection pot, rather than separate ones, could reduce emissions equivalent to 396 kg CO₂e, the same as driving an average passenger car for 982 miles [28]. This simple change requires no additional costs and can be implemented immediately across endoscopic units.

Reusable Endoscopic Accessories

The increasing use of disposable endoscopes and accessories over the past two decades has been driven by concerns about infection control and the costs of managing endoscope-related infections [22]. However, lifecycle analyses reveal that single-use endoscopes produce up to 20 times the carbon emissions of reusable alternatives, with 96% of emissions arising during production [8].

Despite these concerns, the actual risk of infection from properly reprocessed reusable scopes remains low [22]. In the UK, no cases of infection transmission due to the reuse of gastroscopes or colonoscopes have been reported [8]. With strict adherence to cleaning protocols, healthcare facilities can safely shift back toward reprocessing and reusing biopsy forceps, snares, and other accessories, significantly reducing greenhouse gas emissions.

Though the adoption of reusable endoscopic accessories is a vital step toward reducing procedural waste and cost, its feasibility varies significantly across regions due to differences in infrastructure, regulatory oversight, and infection control capabilities. In high-income Western countries, structured reprocessing protocols and stringent quality assurance standards support the safe use of reusable biopsy forceps, snares, and valves. In contrast, lower- and middle-income countries (LMICs) in Asia often face limitations in sterilization equipment, staff training, and surveillance systems, which may compromise reprocessing quality and patient safety if not addressed adequately. Considerable variation in cleaning and disinfection practices for reusable accessories has been observed, often driven by inadequate standardization and resource limitations [29]. Conversely, a recent life cycle assessment found that reusable biopsy forceps, when properly maintained, resulted in a 75% reduction in environmental impact compared to single-use alternatives [30]. These findings underscore the need for region-specific protocols and investment in reprocessing infrastructure to safely scale reusable accessory programs in diverse healthcare settings [31].

Digital Transformation and Paperless Documentation

Paper contributes significantly to hospital waste, making up approximately 30% of the total [32]. The environmental impact of paper extends beyond disposal-it includes the energy consumed in its production, printing, and distribution. Transitioning to digital documentation systems, including electronic medical records and digital consent forms, is a practical and sustainable solution [33].

This transition is supported by growing digital engagement among patients, with 71% reportedly comfortable using QR codes and digital platforms [25]. Blockchain technology can further enhance data security, accessibility, and efficiency within electronic systems, reducing the reliance on paper while improving care quality [32]. When paper use is unavoidable, sustainability can be improved by minimizing print volumes, recycling, and using monochrome printing [8].

Case Studies and Practical Insights

Several institutions have demonstrated the real-world benefits of sustainable practices in gastroenterology. Approaches such as the adoption of recyclable materials, implementation of green procedural protocols, and education of healthcare personnel have been shown to significantly reduce waste volume, operational costs, and associated emissions. A notable example is the Mayo Clinic, which manages over 60,000 pounds of waste daily through comprehensive recycling, incineration, and waste segregation programs, successfully reducing landfill contributions by approximately 90% [33].

Similarly, targeted changes in biopsy collection methods and waste handling practices have shown substantial reductions in carbon emissions without added financial burden [34]. A prospective study in Hyderabad found that GI endoscopy (GIE) procedures have a notable environmental impact, averaging 38.45 kg CO₂e per procedure, of which 83% was attributed to patient travel, with significantly lower waste generation (0.504 kg per procedure) compared to Western data [35]. The study highlights the potential for sustainability through reducing repeat procedures, optimizing patient travel, and improving resource efficiency, especially given India's relatively higher hospital waste recycling rate of 25.7%.

A study by Desai et al. (2023) at a tertiary care academic endoscopy unit in the United States found that every 100 GIE procedures generated approximately 303 kg of solid waste and consumed 1980 kWh of energy [6]. Notably, 20% of this waste was potentially recyclable, indicating significant opportunities for waste reduction through improved segregation and recycling practices. In the United Kingdom, the British Society of Gastroenterology (BSG) has launched a comprehensive strategy to promote sustainability in gastroenterology and hepatology [36]. This includes initiatives such as transitioning to paperless operations, decarbonizing financial investments, and critically evaluating the environmental impact of conferences and clinical practices. The BSG's approach emphasizes integrating sustainability into all aspects of professional activities, from research and training to organizational operations.

Furthermore, a study from Portugal demonstrated the effectiveness of targeted interventions in reducing medical waste within endoscopy units [37]. By conducting a four-stage prospective audit, the study achieved a 12.9% reduction in total waste and a 41.9% reduction in regulated medical waste one month after the intervention, with sustained benefits observed at four months. These findings underscore the potential for significant environmental improvements through systematic waste management strategies in gastroenterology settings. Collectively, these studies highlight the real-world benefits of adopting sustainable practices in gastroenterology as well as the promotion of environmentally responsible clinical operations.

Regional Adaptation of Green Gastroenterology Practices in Asia

While guidelines from Western organizations such as the BSG and the European Society of Gastrointestinal Endoscopy (ESGE) offer comprehensive frameworks for sustainability in clinical practice, their direct application in Asia requires contextual adjustments. Recognizing this, the Asia-Pacific Association for Gynecologic Endoscopy and Minimally Invasive Therapy (APAGE) recently released a position statement on green and sustainable endoscopy tailored to Asian healthcare systems, emphasizing low-cost, high-impact strategies including rational procedure utilization, sustainability-focused education, and digital health integration [38].

Healthcare infrastructure and economic diversity across Asia influence how green gastroenterology initiatives can be operationalized. Many countries in the region operate under budgetary constraints that make high-cost investments, such as energy-efficient HVAC systems, water-optimized reprocessing units, or widespread adoption of reusable endoscopes, less feasible compared to Western systems [39-40]. In such settings, environmentally conscious behavioral interventions, like reducing unnecessary procedures, minimizing redundant biopsies, and extending the use of reusable accessories, can achieve meaningful reductions in carbon emissions with minimal additional financial burden. The region also faces significant gaps in medical waste management. Studies from India, Pakistan, and parts of Southeast Asia have documented inadequate segregation of infectious, pharmaceutical, and general healthcare waste [41]. Improper disposal practices contribute to environmental degradation, groundwater contamination, and occupational hazards, underscoring the need for regulatory reform and targeted training in waste segregation, storage, and disposal [42-43]. In the absence of robust national policies or recycling infrastructure, these basic improvements offer the most immediate path toward sustainability.

Interestingly, many Asian countries possess vibrant local medical manufacturing sectors, particularly India, China, and Vietnam, which offer opportunities for regionally adapted solutions. Local procurement of endoscopic devices, especially those with biodegradable packaging or modular reusable components, could shorten supply chains and reduce the carbon emissions associated with international transport and waste [44]. Government support for sustainable product standards in manufacturing could accelerate these transitions and promote circular healthcare economies within the region.

A significant barrier to green gastroenterology adoption in Asia is the limited incorporation of environmental sustainability into medical education and professional training. A multinational survey found that environmental health remains an underemphasized topic across most Asian teaching hospitals [45]. This knowledge gap reduces clinician awareness of the ecological consequences of clinical decisions. Embedding sustainability content into medical school curricula, fellowship training, and continuing medical education programs would equip healthcare professionals with the tools needed to implement environmentally responsible practices in endoscopy units [46].

Despite infrastructural and educational limitations, Asia is uniquely positioned to leverage digital transformation as a driver of sustainability. Countries such as India, Singapore, and Indonesia have rapidly scaled mobile health technologies and teleconsultation platforms, creating an enabling environment for the expansion of virtual endoscopy follow-ups, remote procedural training, and paperless documentation. These digital tools significantly reduce emissions associated with travel and logistics while enhancing equitable access to specialized care [47].

A comparison of existing frameworks further illustrates the importance of regional customization (Table 1). The ESGE promotes a "5R" strategy - Reduce, Reuse, Recycle, Rethink, and Research - that offers a structured and evidence-based model but requires strong infrastructure and administrative coordination [43]. In contrast, the BSG's strategy emphasizes decarbonization across institutional governance, procurement, and research, making it ideal for high-resource settings with integrated sustainability targets [36]. APAGE, however, focuses on pragmatic, context-sensitive actions, such as triaging procedures, prioritizing reusable tools where safe, and encouraging hybrid education formats, making it more feasible in resource-constrained healthcare systems [38].

**Table 1:** Comparative overview of sustainability frameworks from major gastroenterology associations ESGE- European Society of Gastrointestinal Endoscopy; BSG- British Society of Gastroenterology; APAGE- Asia-Pacific Association for Gynecologic Endoscopy; LMICs- lower and middle income countries

Organization	Focus	Strength	Limitation
BSG [36]	Institutional transformation and decarbonization	Broad policy integration	Less specific for Asia
APAGE [38]	Contextual clinical practice change	Practical for LMICs	Still evolving; less widely adopted
ESGE [43]	5R (Reduce, Reuse, Recycle, Rethink, Research)	Structured and evidence-based	May require robust infrastructure

Although Asia faces multiple implementation challenges, several promising innovations are emerging. In Japan and South Korea, endoscopy units are trialing capsule-based diagnostics and optimized sterilization pathways, while hybrid conference formats and tele-endoscopy initiatives have gained traction in Singapore, China, and India [21,36,40].

To move forward, regional gastroenterology societies should prioritize the integration of green practices into postgraduate and CME training. A simplified 3R model-Reduce, Reuse, Rationalize-could serve as a feasible adaptation of ESGE's more comprehensive framework for low- and middle-income countries. Pilot projects evaluating the reuse of endoscopic accessories, triage algorithms to avoid unnecessary procedures, and virtual training platforms should be encouraged in high-volume institutions. Finally, cross-border collaboration between APAGE, ESGE, and national GI societies could facilitate the development of lifecycle assessment tools, regional emission benchmarks, and shared policy templates to support sustainable gastroenterology practice throughout Asia.

Greener Pharmaceutical and Chemical Use

Endoscopic procedures are essential in gastroenterology, yet the disinfection and sterilization processes, especially for reusable instruments, pose significant environmental and occupational safety concerns (Table 2). Disinfection and reprocessing of endoscopes and their accessories are central to infection prevention but also contribute significantly to the environmental footprint of gastrointestinal services. High-level disinfectants such as glutaraldehyde, peracetic acid, and ortho-phthalaldehyde (OPA) are widely used but pose risks to both the environment and healthcare personnel through chemical emissions and occupational exposure. A recent study found that glutaraldehyde-based disinfectants, commonly used for flexible endoscopes, release volatile organic compounds (VOCs) and generate hazardous waste that requires specialized handling and disposal systems, which are inconsistently implemented in many Asian facilities [48]. The choice and handling of disinfectants can substantially influence both safety and sustainability outcomes. Emerging alternatives such as hydrogen peroxide-based systems or low-temperature plasma sterilization offer lower toxicity and shorter reprocessing times, though they remain underutilized in low-resource settings due to higher initial costs [49]. Furthermore, automated endoscope reprocessors (AERs), while efficient and water-saving in high-income countries, are less accessible in LMICs, necessitating the development of context-appropriate protocols that balance infection control with ecological responsibility [50]. A shift toward validated, low-toxicity disinfectants, combined with reprocessing audit systems, could enhance both environmental and patient safety in endoscopic practice.

**Table 2:** Comparative evaluation of endoscopic disinfection methods

Method	Efficacy	Environmental Impact	Limitations
Ethylene oxide (EO)	Moderate	High (toxic residue)	Leaves tissue debris [51]
Autoclaving	High	Low	Best for complex instruments [51]
Plasma-activated gas (PAG)	High (biofilm disruption)	Low (biodegradable)	Safe ozone dissipation [52]
Ultraviolet C (UVC) light	Moderate (70% reduction)	Low	Best for surfaces, not devices [53]

Traditional high-level disinfectants and methods like ethylene oxide (EO) gas sterilization consume considerable resources and produce hazardous chemical waste [51]. Recent advancements have explored more sustainable alternatives, including the use of argon plasma-activated gas (PAG), which has demonstrated a high degree of efficacy in reducing viable bacterial counts and disrupting 24- and 48-hour biofilms within endoscopic channels, all while minimizing structural damage to equipment [52]. Notably, ozone levels generated during plasma exposure return to safe concentrations within 30 seconds, enhancing safety for healthcare personnel and patients alike. This positions PAG as a promising low-residue and biodegradable disinfectant option, particularly suited to high-throughput environments demanding both efficiency and safety [52]. Complementing this approach, ultraviolet C (UVC) light disinfection systems have been tested for decontaminating stainless steel surfaces tainted with pathogens such as methicillin-resistant *Staphylococcus aureus *(MRSA), *Klebsiella pneumoniae*, and *Clostridium difficile *[53]. Although UVC exposure achieved an approximate 70% reduction in bacterial load, manual cleaning still left 50% of surfaces with detectable contamination, indicating UVC's optimal use lies in targeted sanitation of high-touch surfaces rather than complex instrument interiors [54]. When comparing sterilization methods for reusable biopsy forceps, EO gas sterilization was found to leave trace *Escherichia coli* in 3.3% of instruments and residual organic debris visible under electron microscopy [55]. In contrast, autoclaving achieved complete microbial elimination, establishing its superiority for sterilizing intricately designed tools. Collectively, these findings advocate for a more nuanced approach to sterilization, balancing environmental considerations with clinical safety by leveraging novel technologies like PAG and UVC where applicable, while maintaining autoclaving as the gold standard for reusable instruments.

Beyond disinfection, attention must also be directed toward pharmaceutical use in gastroenterology, particularly the overprescription of proton pump inhibitors (PPIs) and antibiotics, which presents dual threats of antimicrobial resistance and environmental degradation [56]. A substantial proportion of these medications is excreted unmetabolized, entering soil and water systems and altering microbial ecosystems, thereby promoting drug-resistant strains [56]. Moreover, the industrial processes underlying pharmaceutical manufacturing contribute heavily to carbon emissions and resource consumption. To address this, antibiotic stewardship programs have emerged as effective strategies within gastroenterology, particularly in the management of conditions like Clostridium difficile infection, spontaneous bacterial peritonitis, and Helicobacter pylori treatment [54,57]. These initiatives not only improve therapeutic outcomes but also curb environmental contamination. Concurrently, green pharmacy movements are gaining traction, advocating for sustainable drug manufacturing practices, the use of biodegradable packaging materials, and the minimization of pharmaceutical waste [58]. When coupled with evidence-based prescribing protocols and clinician-patient education, these strategies lay the groundwork for a more ecologically responsible approach to medical care.

Reducing Travel-Related Emissions

Medical conferences play a pivotal role in advancing gastroenterological research and collaboration, yet they are substantial contributors to carbon emissions, largely due to air travel. A 2023 study estimated that a single neuroscience conference generated over 17,000 metric tons of CO₂, highlighting the significant environmental toll of academic gatherings [59]. Although specific data on gastroenterology events such as Digestive Disease Week (DDW) or United European Gastroenterology (UEG) Week is limited, these large-scale, globally attended conferences likely produce comparable emissions. The COVID-19 pandemic acted as a catalyst for the adoption of virtual and hybrid conference models, which have proven to significantly lower emissions while enhancing global accessibility [60]. Increasingly, gastroenterology societies are embracing multi-hub events, where regional centers are digitally connected, effectively reducing the need for long-haul travel without compromising the exchange of knowledge.

In parallel, the evolution of remote endoscopy training has further contributed to emission reduction within the field. Virtual simulation platforms and real-time proctoring technologies allow expert clinicians to supervise procedures, deliver feedback, and conduct case-based discussions remotely [61]. These tools not only reduce the carbon footprint associated with faculty travel but also democratize access to advanced training in geographically or economically underserved regions. While some studies indicate that in-person conference attendees may enjoy higher academic visibility and h-index scores, hybrid models offer a pragmatic compromise, retaining academic rigor while aligning with sustainability imperatives [62,63]. Moving forward, the integration of sustainability metrics into the planning of educational events will be essential as professional societies seek to balance innovation with environmental stewardship.

Telemedicine represents another impactful avenue for reducing travel-related emissions in gastroenterology. Virtual consultations decrease the need for patient and provider travel, thereby reducing greenhouse gas emissions linked to personal vehicle use and facility energy demands. Numerous studies have validated the effectiveness of telehealth for managing conditions such as gastroesophageal reflux disease (GERD), inflammatory bowel disease (IBD), and routine post-colonoscopy follow-ups, with comparable patient satisfaction and clinical outcomes [64]. Embedding telemedicine into standard outpatient care, particularly for stable chronic conditions, offers a scalable, low-emission alternative to traditional models of care [65]. As digital health tools continue to evolve, their deliberate integration into gastroenterology practice can serve both clinical and planetary health goals.

Barriers to implementing sustainable practices

Despite growing awareness of healthcare's environmental impact, the integration of sustainable practices into gastroenterology faces several persistent barriers, ranging from economic constraints and regulatory hurdles to behavioral resistance within clinical teams (Table 3). These challenges complicate the adoption of greener technologies and workflows, slowing progress toward a low-carbon, climate-smart healthcare system.

**Table 3:** Summary of key barriers to implementing sustainable practices in gastroenterology

Barrier category	Key Issues	Reference(s)
Economic constraints	- High upfront costs for reusable instruments and energy-efficient systems. - Limited financial justification due to lack of lifecycle cost data. - Sterilization equipment and training costs may be unaffordable in low-resource settings.	[66-67]
Regulatory uncertainty	- Strict hygiene standards discourage reusable devices. - Infection risks from pathogens like C. difficile and P. aeruginosa drive preference for disposables. - Absence of integrated safety–sustainability guidelines.	[68-69]
Behavioral resistance	- Workflow disruption due to sterilization steps. - Increased workload and documentation needs. - Lack of awareness among staff; sustainability not embedded in clinical culture.	[70-71]
Institutional challenges	- Weak leadership support for green transitions. - Sustainability committees and incentives are not universally implemented. - Training and education programs are inconsistently available.	[71]

To address these challenges, a growing number of hospitals have implemented best practices centered around staff engagement and policy incentives. Educational campaigns that emphasize the link between healthcare operations and climate change have proven effective in shifting attitudes and behaviors. For example, integrating sustainability into staff onboarding and continuing education has increased awareness and encouraged ownership of eco-friendly practices across departments. Some institutions have also introduced performance-based incentives or recognition programs that reward departments for reducing waste or meeting emission reduction targets [71]. At the administrative level, cross-disciplinary sustainability committees, including clinicians, infection control experts, and procurement officers, can help develop balanced policies that align environmental goals with safety and efficiency requirements.

Standardized metrics for healthcare sustainability, funding for green infrastructure, and clearer guidance from accreditation agencies can all help align incentives and reduce ambiguity. As the healthcare sector increasingly acknowledges its responsibility in the climate crisis, gastroenterology departments must be empowered with the tools, training, and institutional support needed to overcome these systemic barriers.

Future directions and recommendations

As the climate crisis intensifies, the imperative to decarbonize healthcare, including gastroenterology, has never been more urgent. While several promising interventions are emerging, scaling these solutions across clinical, institutional, and policy levels will be essential to achieving meaningful environmental impact. Table 4 summarizes these key areas, offering a structured overview of actionable recommendations that align with current evidence and address both global and regional challenges in implementing sustainable gastroenterology.

**Table 4:** Future directions and recommendations for sustainable gastroenterology practice

Focus area	Recommended actions	Reference(s)
Sustainability metrics and tools	Develop procedure-specific lifecycle assessment (LCA) tools for gastroenterology. Integrate environmental metrics into clinical decision-making and electronic health record (EHR) systems.	[72]
Education and professional training	Embed sustainability content in accreditation and continuing medical education (CME) programs. Use simulation and decision-support tools to promote low-waste, reusable practices.	[73]
Technology and innovation	Promote tele-endoscopy, AI tools, and circular device design. Invest in biodegradable disinfectants, energy-efficient sterilizers, and digital emissions tracking.	[74]
Policy and governance	Adopt green procurement standards, product carbon labeling, and climate-linked reimbursement. Offer tax incentives and grants for sustainable innovations.	[75-76]
Equity and global collaboration	Adapt sustainability strategies to low- and middle-income countries (LMIC) contexts. Establish cross-border platforms for technology transfer and joint protocol development.	[76]

It is important to acknowledge that the current literature on sustainability in gastroenterology is evolving and remains constrained by limited high-quality comparative studies, inconsistent reporting of environmental outcomes, and the absence of standardized sustainability metrics. As a result, precise cross-institutional comparisons and formal cost-benefit analyses remain challenging. Future research should prioritize the development of validated lifecycle assessment methodologies and consensus-driven reporting standards to strengthen the evidence base. 

## Conclusions

In light of the escalating climate crisis, gastroenterology must evolve to integrate environmentally responsible practices that align with the broader goals of sustainable healthcare. This review highlights the substantial carbon footprint associated with endoscopic procedures, pharmaceutical use, medical waste, and travel-related activities within the specialty. Encouragingly, a variety of practical strategies, ranging from the adoption of reusable endoscopic accessories and eco-friendly disinfectants to the promotion of telemedicine, virtual conferences, and optimized procedural triage, have demonstrated the potential to reduce environmental harm without compromising patient care.

However, implementation is hindered by economic, regulatory, and cultural barriers that require targeted education, policy incentives, and systemic support. Moving forward, sustainability must be embedded in the design of clinical protocols, training programs, and institutional policies. Investment in green technologies, standardized environmental metrics, and collaborative frameworks will be essential to scale solutions across diverse healthcare settings. Ultimately, a greener gastroenterology is not only feasible but necessary, offering an opportunity for the specialty to lead in climate-conscious innovation while upholding its commitment to patient safety, equity, and global health. By taking proactive steps today, the field can contribute meaningfully to planetary health and ensure a more resilient and responsible healthcare future.
